# A Comparison of Co-methylation Relationships Between Rheumatoid Arthritis and Parkinson's Disease

**DOI:** 10.3389/fnins.2018.01001

**Published:** 2019-01-10

**Authors:** Guoping Tang, Hongzhi Pan, Liangde Xu, Rennan Feng, Yongshuai Jiang, Fanwu Kong, Simeng Hu

**Affiliations:** ^1^The Fourth Affiliated Hospital, Zhejiang University School of Medicine, Jinhua, China; ^2^Collaborate Research Center, Shanghai University of Medicine and Health Sciences, Shanghai, China; ^3^College of Bioinformatics Science and Technology, Harbin Medical University, Harbin, China; ^4^Department of Nutrition and Food Hygiene, Public Health College, Harbin Medical University, Harbin, China; ^5^Department of Nephrology, The Second Affiliated Hospital, Harbin Medical University, Harbin, China

**Keywords:** Parkinson's disease (PD), rheumatoid arthritis, DNA methylation, Pearson's correlation coefficients, disease

## Abstract

Rheumatoid arthritis (RA) is a complex autoimmune disease. Recent studies have identified the DNA methylation loci associated with RA and found that DNA methylation was a potential mediator of genetic risk. Parkinson's disease (PD) is a common neurodegenerative disease. Several studies have indicated that DNA methylation levels are linked to PD, and genes related to the immune system are significantly enriched in PD-related methylation modules. Although recent studies have provided profound insights into the DNA methylation of both RA and PD, no shared co-methylation relationships have been identified to date. Therefore, we sought to identify shared co-methylation relationships linked to RA and PD. Here, we calculated the Pearson's correlation coefficient (PCC) of 225,239,700 gene pairs and determined the differences and similarities between the two diseases. The global co-methylation change between in PD cases and controls was larger than that between RA cases and controls. We found 337 gene pairs with large changes that were shared between RA and PD. This co-methylation relationship study represents a new area of study for both RA and PD and provides new ideas for further study of the shared biological mechanisms of RA and PD.

## Introduction

Rheumatoid arthritis (RA) is a chronic autoimmune disease (Adkar et al., 2017; Nair et al., 2017; Choudhary et al., 2018; Liu and Page, 2018; Mizoguchi et al., 2018) with multiple environmental risk factors, including lifestyle factors, hormones, infections, and smoking, as well as interactions between genes and the environment (Klareskog et al., 2009; Scott et al., 2010; Karlson and Deane, 2012; Aleyd et al., 2016; Choudhary et al., 2018; Soulaidopoulos et al., 2018). Parkinson's disease (PD) (Antony et al., 2013; Goldman, 2014), like Alzheimer's disease (AD) (Zhou et al., 2018a), is a common neurodegenerative disease. The most important pathological change of PD is the degeneration of dopamine (DA) neurons in the midbrain, which causes a significant decrease in the striatum DA content and results in disease (Antony et al., 2013). The exact cause of this pathological change is still unclear. Genetic factors, environmental factors, aging, and oxidative stress may be involved in the degenerative death of PD dopaminergic neurons (Goldman, 2014).

In recent years, many studies have paid close attention to epigenetic mechanisms (Cui et al., 2018), especially DNA methylation (Liu et al., 2013; Davegårdh et al., 2018; Meehan et al., 2018). Epigenomic differences can represent phenotypic differences resulting from environmental exposure (Viatte et al., 2013). Increasingly, DNA methylation has been investigated as a potential diagnostic biomarker (Nair et al., 2017). DNA methylation occurs when a methyl group is added to cytosine DNA nucleotides to form 5-methylcytosine. This process is catalyzed by methyltransferases (Nair et al., 2017). A large number of studies have shown that DNA methylation can cause changes in chromatin structure, DNA conformation, DNA stability and DNA-protein interactions, to control gene expression (Hannon et al., 2016; Rao et al., 2018).

In 2013, Liu et al. analyzed the genome-wide DNA methylation differences in peripheral blood leukocytes (PBLs) from RA patients and normal controls (Liu et al., 2013). They applied the Causal Inference Test, with genotype as a causal factor, DNA methylation as a potential mediator and RA as the outcome, to identify the differential methylation positions (DMPs) associated with the RA phenotype. They found 264 unique single nucleotide polymorphisms (SNPs) and nine unique DMPs, comprising 535 SNP-DMP pairs, within the major histocompatibility complex region. Those SNP-DMP pairs represent potential methylation-mediated relationships between SNPs and RA disease risk, revealing that DNA methylation is a potential mediator of genetic risk. Many studies have indicated there are strong associations between neurodegenerative diseases and autoimmune diseases (Zhou et al., 2018b). In 2017, Chuang et al. found that PD status had a profound association with DNA methylation levels in blood and saliva, and three out of six PD-related CpG clusters in blood were significantly enriched for genes related to the immune system (Chuang et al., 2017).

Although recent studies have provided profound insights into the DNA methylation of RA and PD, they have not considered the interaction relationships between genes. RA and PD, as complex diseases, may be caused by the interaction of multiple genes (Yi et al., 2017). Based on this concept, in this study we analyzed one-to-one gene co-methylation relationships to further dissect common molecular mechanisms between RA and PD.

## Materials and Methods

### DNA Methylation Data for RA

In this study, we obtained RA methylation data (GSE42861) from the NCBI GEO database. The platform was GPL13534 (Illumina HumanMethylation450 BeadChip, including 485,577 probes). GSE42861 contains 354 RA samples and 335 normal control samples. Bisulphite converted DNA was from PBLs. The matrix file of GSE42861 includes the information for samples and normalized beta values (from 0 to 1) for each sample.

### DNA Methylation Data for PD

We also obtained PD methylation data (GSE111629) from the NCBI GEO database. The platform was also GPL13534. The matrix file includes 572 samples (335 PD samples and 237 normal control samples from whole-blood DNA) and normalized beta values (from 0 to 1) for each sample.

### Mapping of Methylation Positions to Genes

In this study, we analyzed genes on a one-to-one basis to compare the global co-methylation landscapes. We used the same data processing for RA and PD. We mapped 485,577 probes in the GPL13534 platform to 21,225 genes. If there were multiple probes in a gene, we calculated the average beta value of those probes as the methylation value of the gene. The final RA dataset used for subsequent analysis consisted of 21,225 genes and 689 samples (including 354 RA samples and 335 normal control samples), and the final PD dataset included 21,225 genes, 335 PD samples, and 237 normal control samples.

### The Co-methylation Coefficient

We used the Pearson's correlation coefficients (PCC) to measure the co-methylation relationships between gene pairs. We defined the PCC as:

$$\text{r}\left(\text{i},\text{j}\right)\text{=}\frac{\text{1}}{\text{n }-1}\sum_{\text{k}=1}^{\text{n}}\left(\frac{{\text{b}}_{\text{i},\text{k}}{-\bar{\text{b}}}_{\text{i}}}{{\text{S}}_{\text{i}}}\right)\left(\frac{{\text{b}}_{\text{j},\text{k}}{-\bar{\text{b}}}_{\text{j}}}{{\text{S}}_{\text{j}}}\right)$$

where *r*(i,j) is the PCC between gene i and gene j; n is the number of samples; b_i, k_ is the methylation beta value of gene i in sample k; b_i, k_ is the methylation beta value of gene j in sample k; b_i_ is the mean of the methylation beta value of gene i; b_j_ is the mean of the methylation beta value of gene j; S_i_ is the standard deviation (SD) of the methylation beta value of gene i; and S_j_ is the SD of the methylation beta value of gene j. When *r*(i,j) >0, gene i and gene j represent a positive correlation, that is, gene i and gene j are highly co-methylated. When the *r*(i,j) < 0, gene i and gene j represent a negative correlation. In other words, the methylation levels of gene i and gene j are exactly opposite.

## Results

### Comparison of the Distribution of the PCC Between RA and PD

**Figure 1:**
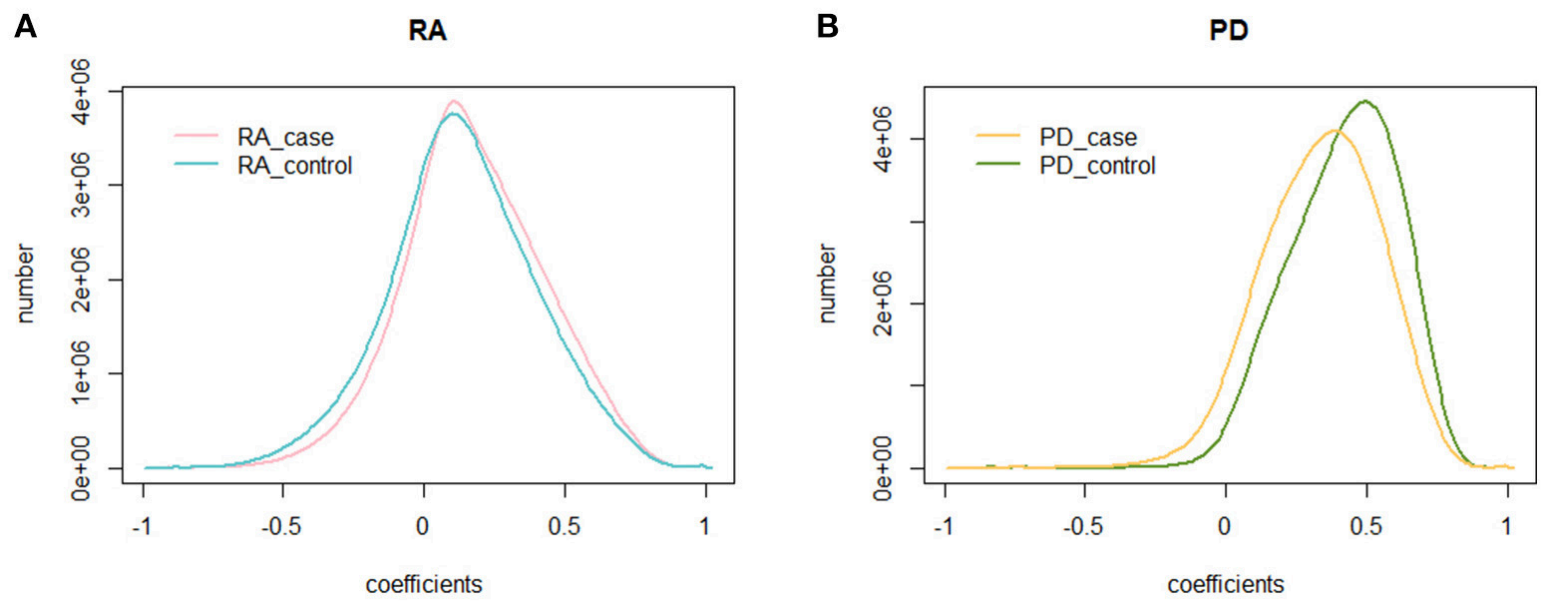
The distribution of the PCC. **(A)** The distribution of the PCC in RA cases and controls. The blue curve is the distribution of the PCC for RA controls, while the pink curve is the distribution of the PCC for RA cases; **(B)** The distribution of the PCC in PD cases and controls. The green curve is the distribution of the PCC for PD controls, while the yellow curve is the distribution of the PCC for PD cases.

We calculated the PCC of 225,239,700 gene pairs for RA cases and RA controls. The mean PCC of the RA cases was 0.174 and the SD was 0.250, while the mean PCC of the RA controls was 0.132 and the SD was 0.260. We then analyzed their distribution and plotted them on a graph (Figure 1A). Both the RA cases and controls showed a similar unimodal distribution of the global PCC.

We also calculated the PCC for the dataset of PD cases and controls. The mean PCC of the PD cases was 0.334 and the SD was 0.209, while the mean PCC of the PD controls was 0.414 and the SD was 0.194. Their distribution is shown in Figure 1B, where the global change of PD cases and controls is larger than that of RA cases and controls.

### Identifying Large Change Gene Pairs for RA

We divided the PCC into eight intervals, named strongest negative correlation (−1 to −0.75), strong negative correlation (−0.75 to −0.5), weak negative correlation (−0.5 to −0.25), weakest negative correlation (−0.25 to 0), weakest positive correlation (0 to 0.25), weak positive correlation (0.25 to 0.5), strong positive correlation (0.5 to 0.75), and strongest positive correlation (0.75 to 1). For each interval of the RA controls, we analyzed the changes in the co-methylation relationship from controls to cases. The results are presented in Table 1.

**Table 1:** The number of gene pairs in eight sections for RA cases and controls.

**Interval**	**RA_case**							
	**[−1, −0.75)**	**[−0.75, −0.5)**	**[−0.5, −0.25)**	**[−0.25, 0)**	**[0, 0.25)**	**[0.25, 0.5)**	**[0.5, 0.75)**	**[0.75, 1]**
	77,481	27,809	80	2	0	0	0	0
RA_control_[−1, −0.75)	(73.53091%)	(26.39126%)	(0.075921%)	(0.001898%)				
	7,299	7,64,774	12,86,473	68,700	108	5	0	0
RA_control_[−0.75, −0.5)	(0.34310%)	(35.94946%)	(60.47277%)	(3.229356%)	(0.005077%)	(0.000235%)		
	2	1,82,660	56,96,562	73,98,531	4,82,134	956	13	0
RA_control_[−0.5, −0.25)	(0.00001%)	(1.32739%)	(41.39685%)	(53.76504%)	(3.503662%)	(0.006947%)	(0.000094%)
	0	1,753	17,84,109	272,23,563	206,88,414	6,50,691	716	0
RA_control_[−0.25, 0)		(0.00348%)	(3.543467%)	(54.06945%)	(41.08982%)	(1.292355%)	(0.001422%)	
	0	2	26,663	82,65,509	596,94,607	192,89,656	2,45,055	50
RA_control_ [0, 0.25)		(0.000002%)	(0.030464%)	(9.443971%)	(68.20562%)	(22.03970%)	(0.279994%)	(0.000057%)	
	0	0	30	50,725	82,54,707	359,08,516	81,84,266	3,747
RA_control_[0.25, 0.5)			(0.000057%)	(0.096800%)	(15.75266%)	(68.52510%)	(15.61824%)	(0.007150%)
	0	0	0	11	15,739	32,31,110	135,39,841	702,925
RA_control_[0.5, 0.75)				(0.000063%)	(0.089990%)	(18.47444%)	(77.41641%)	(4.019097%)
	0	0	0	0	0	577	408,604	10,74,525
RA_control_[0.75, 1]						(0.038889%)	(27.53942%)	(72.42170%)

For RA, there were five gene pairs (*ARHGAP17* and *TSHZ3, DDX54* and *TSHZ3, GATA3* and *GRB2, GRB2* and *TSHZ3*, and *PIGC* and *SHZ3*) where the PCC changed from a strong negative correlation to a weak positive correlation, and 13 gene pairs where the PCC changed from a weak negative correlation to a strong positive correlation. These 18 gene pairs crossed four intervals and recorded significant changes. There was also a total of 1,893 gene pairs crossing no less than three intervals with large changes of the co-methylation relationship from controls to cases. These are shown in Supplementary File S1.

### Identifying Large Change Gene Pairs for PD

For PD, we used the same method to divide the PCC into eight intervals, and changes of the co-methylation relationship from controls to cases are shown in Table 2.

**Table 2:** The number of gene pairs in eight sections for PD cases and controls.

**Interval**	**PD_case**							
	**[−1, −0.75)**	**[−0.75, −0.5)**	**[−0.5, −0.25)**	**[−0.25, 0)**	**[0, 0.25)**	**[0.25, 0.5)**	**[0.5, 0.75)**	**[0.75, 1]**
	53,560	7,274	0	0	0	0	0	0
PD_control_[−1, −0.75)	(88.04287%)	(11.95713%)						
	6,062	58,463	4,277	9	2	0	0	0
PD_control_[−0.75, −0.5)	(8.80938%)	(84.95924%)	(6.21540%)	(0.01308%)	(0.00291%)			
	3	24,024	85,156	16,583	314	5	1	0
PD_control_[−0.5, −0.25)	(0.00238%)	(19.05366%)	(67.53803%)	(13.15213%)	(0.24904%)	(0.00397%)	(0.00079%)	
	9	16,305	4,84,685	20,12,234	11,68,009	12,571	33	0
PD_control_[−0.25, 0)	(0.00024%)	(0.44141%)	(13.12142%)	(54.47531%)	(31.62040%)	(0.34032%)	(0.00089%)	
	6	5,546	3,93,274	73,60,253	298,46,001	54,38,956	33,073	57
PD_control_[0, 0.25)	(0.00001%)	(0.01287%)	(0.91295%)	(17.08621%)	(69.28497%)	(12.62608%)	(0.07678%)	(0.00013%)
	0	594	73,877	22,10,173	293,93,376	586,80,360	54,10,454	4,552
PD_control_ [0.25, 0.5)		(0.00062%)	(0.07714%)	(2.30771%)	(30.69055%)	(61.27001%)	(5.64922%)	(0.00475%)
	0	13	1,807	1,11,235	28,05,248	334,91,914	406,51,108	675,825
PD_control_[0.5, 0.75)		(0.00002%)	(0.00232%)	(0.14309%)	(3.60863%)	(43.08354%)	(52.29303%)	(0.86937%)
	0	0	2	60	3,064	85,461	28,28,820	17,85,012
PD_control_[0.75, 1]			(0.00004%)	(0.00128%)	(0.06516%)	(1.81738%)	(60.15670%)	(37.95944%)

From the PD controls to the PD cases, 15 gene pairs recorded the largest changes of the co-methylation relationship, crossing five intervals. These included two gene pairs (*HIST1H2BH* and *NFKBIE*, and *HIST1H3F* and *NFKBIE*) where the PCC changed from the strongest positive correlation to a weak negative correlation, and 13 gene pairs where the PCC changed from a strong positive correlation to a strong negative correlation. Furthermore, 2,468 gene pairs crossed four intervals, including one gene pair from a weak negative correlation to a strong positive correlation, six gene pairs from the weakest positive correlation to the strongest negative correlation, 594 gene pairs from a weak positive correlation to a strong negative correlation, 1,807 gene pairs from a strong positive correlation to a weak negative correlation, and 60 gene pairs from the strongest positive correlation to the weakest negative correlation.

In total, 196,311 gene pairs crossed no less than three intervals in PD, shown in Supplementary File S2. There was obviously a larger difference in the partial co-methylation relationship between controls and cases in PD than in RA.

### Shared Gene Pairs Between RA and PD

To identify the genetic mechanisms shared in both RA and PD, we extracted the gene pairs that changed across no less than three intervals. We extracted 1,893 gene pairs from RA and 196,311 gene pairs from PD. We found there were 337 gene pairs (in Supplementary File S3) shared between RA and PD. We extracted 28 gene pairs with an absolute value of difference >0.8 from RA and 4,748 gene pairs from PD. Nine of these gene pairs (shown in Table 3) were shared in RA and PD, and these nine gene pairs were included in the 337 gene pairs extracted above.

**Table 3:** Nine gene pairs were shared between RA and PD.

**Gene1**	**Gene2**	**RA**		**PD**	
		**Case_corr**	**Control_corr**	**Case_corr**	**Control_corr**
ARHGAP17	TSHZ3	0.382	−0.561	0.384	−0.497
CDK20	GRB2	0.481	−0.484	0.517	−0.492
CDK20	PLEKHF2	0.458	−0.342	0.492	−0.408
CRY1	GRB2	0.599	−0.252	0.552	−0.264
ETV1	GRB2	0.368	−0.473	0.63	−0.237
GATA3	GRB2	0.279	−0.544	0.59	−0.64
GRB2	LCA5	0.505	−0.36	0.469	−0.41
GRB2	PVR	0.692	−0.32	0.698	−0.114
GRB2	TSHZ3	0.441	−0.616	0.397	−0.588

### Gene Ontology and KEGG Pathway Analysis of Shared Gene Pairs

To further analyze the functional characteristics of the shared gene pairs, we extracted 220 unique genes from the 337 gene pairs by removing duplicate genes, and then annotated them to GO (Gene Ontology) categories and KEGG pathways using DAVID. This found 70 GO categories and four KEGG pathways that were significantly (*P* < 0.05) annotated. Among them, 11 immune-related GO categories included GO: 0002250: adaptive immune response, GO: 0050853: B cell receptor signaling pathway, GO: 0006952: defense response, GO: 0031295: T cell co-stimulation, GO: 0006955: immune response, GO: 0042832: defense response to protozoan, GO: 0030183: B cell differentiation, GO: 0050900: leukocyte migration, GO: 0070671: response to interleukin-12, GO: 0019815: B cell receptor complex and GO: 0001772: immunological synapse. Two GO categories related to brain development were GO: 0030901: midbrain development and GO: 0022029: telencephalon cell migration. In addition, these genes were also annotated into two immune-related KEGG pathways (hsa04662: B cell receptor signaling pathway and hsa05340: primary immunodeficiency). As discussed above, 220 unique genes from the 337 gene pairs play important roles in autoimmune regulation and brain development.

## Discussion

In summary, we calculated the PCC of each gene pair and compared the distribution of the global PCC between cases and controls. We found a similar distribution between cases and controls. Nevertheless, we still found 1,893 gene pairs in RA and 196,311 gene pairs in PD whose co-methylation relationship changed a great deal. Moreover, there were 337 gene pairs that changed across no less than three intervals, and nine gene pairs with an absolute value of difference >0.8, shared between RA and PD. By Gene Ontology and KEGG pathway analysis, we found 220 unique genes from the 337 gene pairs that play important roles in autoimmune regulation and brain development. There are therefore potential pathogenic and genetic mechanisms shared between RA and PD.

**Figure 2:**
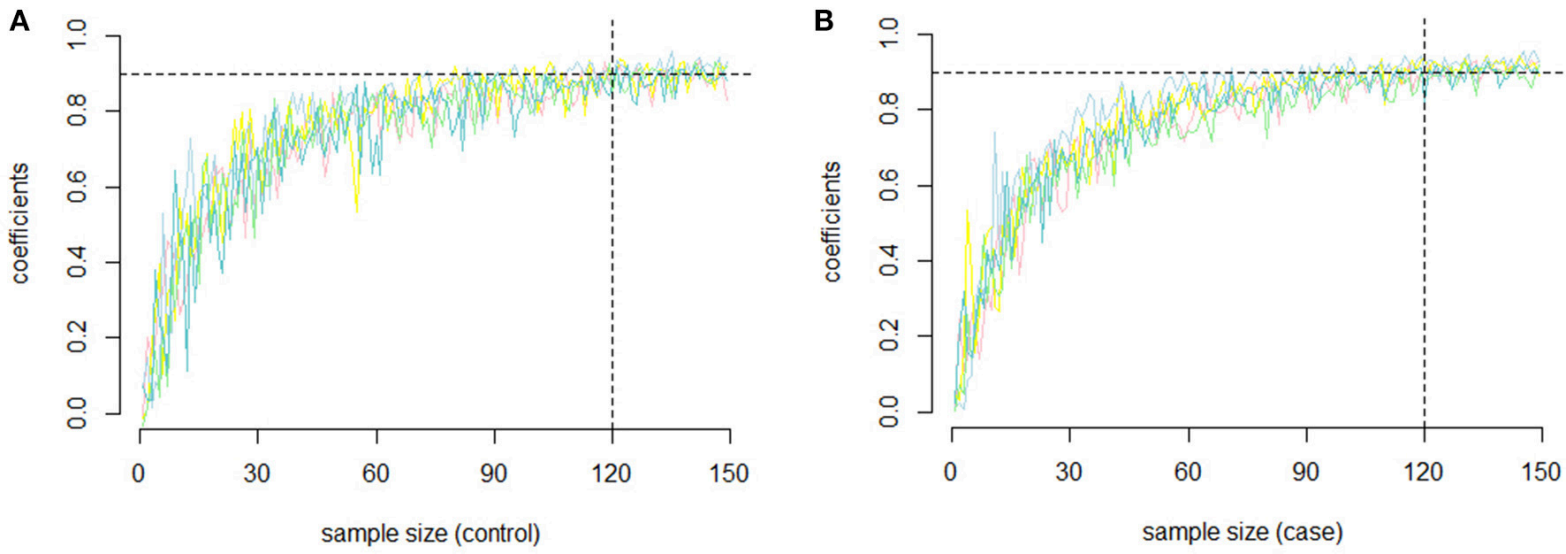
The repeatability of the PCC. **(A)** The relationship between sample size and repeatability of the co-methylation relationships for the five groups of genes in RA cases; **(B)** The relationship between sample size and repeatability of the co-methylation relationships for the five groups of genes in RA normal controls.

In our study, to assess the reliability of the results and the repeatability of the co-methylation relationships, and to determine how many samples are required for a repeat experiment, we randomly selected five groups of genes with 100 genes in each gene group without returning to the samples. We randomly selected samples from each group (the sample size ranged from 2 to 150) and calculated the PCC of each gene pair. To examine the repeatability, we measured the similarity between replicates of the same operator by selecting the same number of samples from the remaining samples and recalculating the PCC. For each of the five groups, the PCC between the first experiment and the second replicate is shown in Figures 2A (RA cases) and **2B** (RA controls). In both RA cases and controls, we found that the PCC increased with increasing sample size. Most of the PCCs were >0.9 when the sample size was >150. In other words, the PCC is repeatable when the sample size is relatively large.

## Author Contributions

SH and RF wrote the main manuscript text. SH, GT, and LX analyzed the data. SH, HP, and FK discussed and improved the article. SH, FK, and YJ conceived and designed the experiments.

### Conflict of Interest Statement

The authors declare that the research was conducted in the absence of any commercial or financial relationships that could be construed as a conflict of interest.

## Supplementary Material

Supplementary File S1 1,893 gene pairs crossing no less than three intervals with large changes of the co-methylation relationship from RA controls to cases.

Supplementary File S2 196,311 gene pairs crossing no less than three intervals with large changes of the co-methylation relationship from PD controls to cases.

Supplementary File S3 337 gene pairs shared between RA and PD.
